# Algorithms for the diagnosis and treatment of restless legs syndrome in primary care

**DOI:** 10.1186/1471-2377-11-28

**Published:** 2011-02-27

**Authors:** Diego Garcia-Borreguero, Paul Stillman, Heike Benes, Heiner Buschmann, K Ray Chaudhuri, Victor M Gonzalez Rodríguez, Birgit Högl, Ralf Kohnen, Giorgio Carlo Monti, Karin Stiasny-Kolster, Claudia Trenkwalder, Anne-Marie Williams, Marco Zucconi

**Affiliations:** 1 Sleep Research Institute, Madrid, Spain; 2 Primary Care Practice, Crawley, Sussex, UK; 3 Somni bene Institute for Medical Research and Sleep Medicine, Schwerin and Neurology Department, University of Rostock, Rostock, Germany; 4 Primary Care Practice, Dusseldorf, Germany; 5 Kings College and University Hospital Lewisham, London, UK; 6 Primary Care Practice, La Alberca, Salamanca, Spain; 7 Department of Neurology, Innsbruck Medical University, Innsbruck, Austria; 8 ReSearch Pharmaceutical Services Inc, Fort Washington, PA, USA; 9 Psychology Department, University of Erlangen-Nuremberg, Nuremberg, Germany; 10 Italian Society of General Practitioners, Florence, Italy; 11 Somnomar, Institute for Medical Research and Sleep Medicine, Marburg and Philipps-University of Marburg, Marburg, Germany; 12 Paracelsus-Elena Hospital, Center of Parkinsonism and Movement Disorders, Kassel, Germany; 13 Medical Education Global Solutions, Paris, France; 14 Sleep Disorders Center, Department of Neurology, San Raffaele Institute, Milan, Italy

## Abstract

**Background:**

Restless legs syndrome (RLS) is a neurological disorder with a lifetime prevalence of 3-10%. in European studies. However, the diagnosis of RLS in primary care remains low and mistreatment is common.

**Methods:**

The current article reports on the considerations of RLS diagnosis and management that were made during a European Restless Legs Syndrome Study Group (EURLSSG)-sponsored task force consisting of experts and primary care practioners. The task force sought to develop a better understanding of barriers to diagnosis in primary care practice and overcome these barriers with diagnostic and treatment algorithms.

**Results:**

The barriers to diagnosis identified by the task force include the presentation of symptoms, the language used to describe them, the actual term "restless legs syndrome" and difficulties in the differential diagnosis of RLS.

**Conclusion:**

The EURLSSG task force reached a consensus and agreed on the diagnostic and treatment algorithms published here.

## Background

Restless legs syndrome (RLS) is a neurological disorder characterised by an irresistible urge to move the legs especially at rest. Symptoms worsen in the evening and night and improve with activity such as walking. RLS may be secondary to, or exacerbated by, a number of conditions that include iron deficiency, pregnancy, end-stage renal disease (ESRD), diabetes and rheumatoid arthritis, or with neurological disorders such as peripheral neuropathy.

As a consequence of sleep disruption and the inability to remain still (including during the daytime, the symptoms of RLS can severely impact on activities of daily living [1].

The main consequences of severe RLS are:

a. Sleep disruption: RLS is the sleep disorder which causes the greatest chronic loss of sleep. Results from several surveys report that most RLS patients slept an average of 5 hours a day [2-5]. Sleep loss by itself causes daytime drowsiness, difficulties concentrating, loss of performance and negatively impacts mood.

b. Difficulties resting and remaining still: this happens predominantly in the evening and at night, but also at other times during the day. Consequently patients have difficulties with work, travelling and social events [1].

Until recently RLS was considered a rare disorder; poor recognition of symptoms, the absence of symptoms during most of the day (with an onset only at night), accompanied with an often "bizarre" description of symptoms, frequently led to the consideration of a psychogenic origin of these symptoms. The absence of any classical objective findings on classical neurological tests--such as nerve conduction studies or electromyography--further contributed to this consideration. Furthermore, whenever RLS patients experience sleep disturbance, they frequently cannot relate their sleep problem to the disturbance of their legs and do not report these symptoms to their physician. As a result, a lack of interest in RLS by the entire medical profession has existed historically. Nevertheless, over the last decades, RLS has emerged not only as a common, but also as a sometimes severe disorder [6]. In 1995 the International RLS Study Group (IRLSSG) established four clinical diagnostic criteria for RLS that were later refined and reviewed during a National Institutes of Health (NIH) workshop in 2002 (see Table 1) [7]. As far as the prevalence of RLS is concerned, adult population studies have been carried out and the majority of those undertaken in Western Europe and North America have shown a prevalence ranging between 3 and 10%.

**Table 1:** Essential diagnostic criteria

Essential criteria	Supportive criteria	Associated features
An urge to move the legs, usually accompanied/caused by uncomfortable/unpleasant sensations in the legs.	Positive family history of RLS.	Natural clinical course of the disorder.
Urge to move or unpleasant sensations begin or worsen during periods of rest or inactivity.	Positive response to dopaminergic drugs.	Sleep disorders are a frequent but unspecific symptom of the RLS.
Urge to move or unpleasant sensations are partially/totally relieved by movement, at least as long as the activity continues.	PLMW/PLMS as assessed with polysomnography or leg activity devices.	Medical evaluation/physical examination: The neurological examination is usually normal.
Urge to move or unpleasant sensations are worse in the evening/night than during the day, or only occur in the evening/night.		Probable causes for secondary RLS should be excluded.

## Methods

Given the high prevalence of RLS, the diagnosis of this disorder should occur principally in the primary care setting. Unfortunately this is not the case as identification of RLS in primary care occurs with substantial difficulties. Furthermore, RLS is mismanaged despite the recent publication of evidence-based guidelines on its treatment [8,9]. The published guidelines rarely address the general practitioner (GP)/primary care physician (PCP), instead they address for the most part neurologists and have tailored management and resources available to experts in neurology, psychiatry or sleep medicine. There are few resources available to the GP to facilitate RLS management. In order for RLS to be appropriately managed from primary care upwards, it is therefore necessary to provide GPs with both diagnostic and treatment guidelines. A previous consensus based-treatment algorithm was published by the Medical Advisory Board of the Restless Legs Foundation in 2004 [10], however, since this time many new randomized-controlled studies have been published that change how RLS should be treated.

In order to tackle emerging difficulties for diagnosing RLS in primary care, the European RLS Study Group http://www.eurlssg.org established a task force consisting of experts and primary care practioners--authors of the current paper--from several European countries with the objective of identifying and overcoming barriers to the diagnosis and treatment of RLS in primary care during three consensus meetings that took place in several European cities over 2008 and 2009. This report summarises the discussions and conclusions of this task force and proposes diagnostic and treatment algorithms to facilitate the diagnosis and treatment of RLS in primary care.

## Results

### Barriers to diagnosis

Despite the high prevalence of RLS and the high percentage of RLS sufferers with symptoms that impact on activities of daily living, RLS remains underdiagnosed and also misdiagnosed--as skin irritation, arthritis, malingering, and venous disorders in adults, and as growing pains or attention deficit hyperactivity disorder (ADHD) in children--which consequently leads to many sufferers having to wait several years before a correct diagnosis is made, this is especially the case for patients who have chronic RLS that began in childhood. In a German population-based survey the overall prevalence of a known doctor diagnosis of RLS was 2.3%, the ratio of diagnosed to undiagnosed RLS was 1:3 [11]. In a French study only 5.3% of RLS sufferers received a diagnosis of RLS despite the fact that 53% of the sample had consulted their doctor with RLS symptoms; 60% of RLS sufferers had received a previous vascular diagnosis mainly related to venous disease [12]. In the REST primary care study performed in the USA and five European countries, 64.8% of sufferers reported consulting a physician about their RLS symptoms, of these only 58% received *any *diagnosis, while 12.9% were given a diagnosis of RLS; the general practitioner reported that only 37.9% of these RLS sufferers had consulted for RLS symptoms [6]. Similar examples of underdiagnosis and mismanagement have been provided by large studies performed in the UK, USA and in Ireland [1,4,13].

### The diagnosis of RLS

A clinical diagnosis of RLS can only be made if patients complain of four key symptoms which constitute the essential criteria defined by the IRLSSG (Table 1 and below) [14,15]. There is no specific biological marker for RLS, however, the diagnostic certainty of these criteria can be improved if supportive clinical criteria, such as a positive levodopa response,[16] periodic limb movements (PLMs),[17] or the presence of a positive family,[7] are present (Table 1). The four essential criteria are:

#### 1. Urge to move the legs or other body parts usually accompanied or caused by unpleasant sensations

It is possible that the patient has an urge to move that is not accompanied by uncomfortable sensations. These sensations appear predominantly in the legs, but the arms, trunk and face [18] can also be affected [19]. The symptoms are often described as being located deep inside the legs, and a sense of movement inside the leg is also evoked. Because the symptoms are unlike usual sensory experiences, patients have difficulties in describing them. In this way, a myriad of terms are used by patients to describe their symptoms: creeping, crawling, itching, burning, tugging, indescribable, aching, like an electric current, restless, painful etc [7,20].

#### 2. Urge to move or unpleasant sensations begin or worsen during rest or inactivity

The urge to move the legs and/or the uncomfortable sensations being with rest, be it sitting or lying down. The physical immobility and decreased central system activity that characterize rest are thought to be implicated in the onset of symptoms [21].

#### 3. Urge to move or unpleasant sensations are partially or totally relieved by movement

Relief from RLS symptoms is seen with activation of the motor system. Symptoms, which can be unilateral or bilateral, may be totally or partially relieved by movement such as walking or stretching but reappear shortly after movement ceases. The more severe the RLS, the more vigorous the movement needs to be. If no relief is seen with movement it is important to ask patients if during the early stages of their RLS, movement relieved symptoms; it is possible that the condition has become so severe that voluntary movement no longer has an effect on symptoms. Counter stimulation such as massaging or hitting the legs can also relieve symptoms.

#### 4. Urge to move or unpleasant sensations are worse in the evening or at night or occur only in the evening or at night

The circadian pattern of symptoms is necessary for a diagnosis of RLS to be confirmed. Symptoms are at their peak in the hours just after midnight and are at their nadir mid- to late-morning [22,23]. This circadian rhythm also corresponds to the circadian decreases of iron availability which may limit dopamine synthesis [24].

### Potential barriers to diagnosis

#### Presentation of symptoms

In general, RLS does not present as a motor-sensitive problem, but through symptoms such as disturbed sleep [25], pain or unspecific increased motor activity. The reason that sleep disturbance is often the reason for consultation is because the circadian pattern of RLS causes difficulty in falling asleep, getting back to sleep [23], and can cause awakenings during the night due to the discomfort in the limbs [26]. Patients' quality of life can also be affected and chronic disruption of sleep or reduced duration of total sleep time can lead to depression, anxiety, cognitive and social dysfunction [26-28].

#### The term "restless legs syndrome"

A major barrier to diagnosing RLS is the language patients use to describe their symptoms (see Table 2), as well as cultural differences that appear when RLS sufferers describe these symptoms. For example, a description of symptoms as resembling "water moving in my legs" does not confer the seriousness and credibility of symptoms.

**Table 2:** Common terms use to describe RLS [7]

**Creepy-crawly**	**Tearing**
Insects/ants crawling	Throbbing
Jittery	Tight feeling
Pulling	Grabbing sensation
Worms moving	Itching bones
Soda bubbling in the veins	Electric current
Electric current	Fidgets
Pain	Twitching
Burning	Water moving
Tingling	Aching

RLS is also called Ekbom Syndrome, but the term RLS has been preferred by the medical community because it is more descriptive. The problem with the term "restless legs syndrome" is that it is a term that is confusing, because it gives the impression that RLS is a lifestyle disorder as opposed to a nosological entity with a genetic basis. Genome-wide association studies have identified gene variants within MEIS1, BTBD9, MAP2K5 and LBOXCOR1 [29]. It lacks the specific relation to a cause of the symptoms and completely remains in the descriptive area.

#### Differential diagnosis and mimics (see table 3)

**Table 3:** Differential diagnosis

Meeting Criteria	Comment		Disorder
**Urge to move & unpleasant sensations in the legs** **Symptoms begin/worsen during periods of rest or inactivity**. **Symptoms relieved with movement** **Symptoms worse in the evening/night**	Definite RLS	Awake symptom diagnosis made by clinical history; uncomfortable urge to move with or without deep creepy-crawling sensation brought on at time of inactivity or rest (sitting or lying); immediate relief either complete or partial with movement; symptomatic relief is persistent as long as movement continues; presence of circadian pattern with peak around midnight and nadir in the morning	**RLS**

**Urge to move **- **Symptoms begin/worsen during periods of rest or inactivity**. **Symptoms relieved with movement** **Orthostatic hypotension**	Neurological disorder with "urge to move"	Feeling of restlessness which may be localized in legs, brought on by sitting still; should not occur while lying down but might be relieved by movement; occurs in patients with orthostatic hypotension	**Hypotensive akathisia**

**Unpleasant sensations in the legs** **Symptoms relieved with movement** **Symptoms worse in the evening/night** **No positive response to dopaminergic drugs**	Pain Disorder	Dysesthesias and pain in the legs, frequently one-sided, often radicular arrangement of sensory symptoms, atrophic changes of musculature, no urge to move the legs, symptoms can be initiated by sitting and lying and improve by movement, usually neurological and neurophysiological deficits, does not respond to dopaminergic therapy	**Radiculopathy**

**Unpleasant sensations in the legs** **Symptoms relieved with movement** **Symptoms worse in the evening/night **-	Vascular Disorder	Dysesthesias and pain in the legs. May appear to occur with or after rest but is associated with or occurs after periods of standing/walking; ntensity increased by movement and usually relieved by prolonging rest often best in a lying position, no urge to move, no circadian pattern, usually no sleep disturbances, frequently associated with skin alterations and edemas. Often associated with vascular disease, circadian pattern if any relates more to activity levels	**Vascular claudication, neurogenic claudication**

**Urge to move** **Symptoms begin/worsen during periods of rest or inactivity** **Periodic limb movements** **History of neuroleptics**	Neurological disorder with "urge to move"	Looks like very severe RLS affecting the whole body -but usually without any sensations of pain reported by RLS patients often no relief with movement;, should have a history of specific medication exposure	**Neuroleptic-induced akathisia**

**Unpleasant sensations in the legs** **Symptoms begin/worsen during periods of rest or inactivity** **No positive response to dopaminergic drugs**	Pain Disorder	Sensory symptoms commonly reported as numbness, burning, and pain; not as common in RLS; numbness is rare in RLS, no urge to move; sensory symptoms usually present throughout the day, less frequent at night, complete and persistent relief is not obtained while walking or during sustained movement	**Neuropathy**
**No periodic limb movements**			

**Unpleasant sensations in the legs** **Symptoms begin/worsen during periods of rest or inactivity**.	Pain Disorder	Patients after surgeries frequently do not remember the origin of their complaints. They almost always report symptoms in the legs or in the back, when lying or sitting or during movement.	**Chronic pain syndrome (lumbal, cervical)**

**Unpleasant sensations in the legs** **Symptoms relieved with movement**	Disorders without "urge to move"	Often comes on with prolonged sitting or lying in the same position but usually relieved by a simple change in position, unlike RLS, which often returns when change of position, movement, or walking is not continued, no circadian pattern	**Positional discomfort**

**Symptoms relieved with movement** **Symptoms worse in the evening/night**	Neurological disorder with "urge to move"	Leg cramps or charley horse cramps can come on at night and are relieved with stretching or walking; no urge to move; experienced as a usually painful muscular contraction, often involving the calf muscles, unlike RLS sensations; sudden onset, occurs not regularly, short duration, usually palpable contractions	**Nocturnal leg cramps**

**Unpleasant sensations in the legs** **Symptoms worse in the evening/night** **Sleep disturbance**	Sleep-related Disorders	Involuntary muscle (myoclonic) twitch which occurs during falling asleep, described as an electric shock or falling sensation which can cause movements of legs and arms. Occurring once or twice per night, frequent in the population.	**Hypnic jerks**

**Unpleasant sensations in the legs** **Symptoms worse in the evening/night** **Sleep disturbance**	Psychiatric Disorders	Depressive disorder with somatic symptoms like psychomotor agitation and diverse somatic complaints, circadian pattern with early awakening in the morning, daytime sleepiness.	**Depression, various forms with somatic syndrome**

**Urge to move** **No positive response to dopaminergic drugs** **No sleep disturbance**	Neurological disorder with "urge to move"	Occurs in subjects who fidget, especially when bored or anxious, but usually do not experience associated sensory symptoms, discomfort, or conscious urge to move; symptoms do not bother the subject, usually lacks a circadian pattern, more of a type of psychic restlessness, less sleep disturbances, no response to dopaminergic medication	**Volitional movements, foot tapping, leg rocking**

**Urge to move** **No positive response to dopaminergic drugs** **No periodic limb movements**	Disorders without "urge to move"	Discomfort centered more in joints, may not have prominent circadian pattern as seen in RLS, increase of symptoms during movement .does not respond to dopaminergics, usually no PLMs	**Arthritis, lower limb**

**Urge to move** **Sleep disturbance**	Disorders without "urge to move"	Multiple, alternating, multiform complaints in muscle groups and joints; sometimes leg-accentuated but mostly whole body affected; frequent sleep disorders, no circadian pattern, no relief by movement, no dopaminergic response	**Fibromyalgia**

**Urge to move**	Vascular Disorder	Discomfort in legs, some relief with massage or inactivity	**Varicose veins**

The diagnosis of RLS necessitates that the physician is aware of the disorder and its variety of symptoms. When there is a lack of awareness about what exactly RLS is, then the probablility of misdiagnosis is more likely. This is especially the case with RLS mimics, which meet the essential diagnostic criteria but do not constitute RLS. Important mimics include peripheral neuropathy, cramps, positional discomfort, akathisia and anxiety disorders [30]. RLS also needs to be differentiated from other conditions that can also coexist with it such as peripheral neuropathy, lower limb pain conditions of different origin, parkinsonism with sensory symptoms or motor fluctuations with dyskinesias etc. Ekbom's description of "irritable legs" underscores that "the paraesthesia is felt in the lower legs (not the feet). It is never experienced superficially in the skin, but deep down in the calf or sometimes the shin) [31]. The high prevalence of concomitant RLS in the Parkinson's disease population may reflect the medication effect, however there may also be mimics or overlap of some PD symptoms with RLS [32,33]. The diagnosis of RLS can be complicated by a number of other conditions as shown in Table 3.

### Diagnostic algorithm

#### 1. Leading symptoms: Insomnia and unpleasant sensations in the legs

As with all diagnostic algorithms there has to be a presenting symptom that alerts the physician to the possible presence of the disorder in question. In reviewing the literature [1,6,34], but also from experience with patients, the task force concluded that the opening questions should concern both insomnia or sleep problems and unpleasant sensations in the legs. Large epidemiological studies have demonstrated that the symptoms with which patients present concern sleep or unpleasant sensations in the legs. In the REST primary care study sleep (sleep-related symptoms, daytime sleepiness) and discomfort in the legs (pain, twitching and jerks, uncomfortable feelings) accounted for the most troublesome symptom for majority of patients [6]. In a general population study more than 75.5% of RLS sufferers report at least one sleep-related problem [1]. Complaints about sleep problems or leg problems as a potential indicator for RLS were investigated by Crochard et al.[34]. In this study a diagnosis of RLS was given to 42.6% of patients with leg complaints, 35.5% of those with sleep complaints, 54.9% of those with both complaints, and 12.9% of those with no complaints.

#### 2. The RLS Diagnostic Index (RLS-DI)

If a patient presents with insomnia/sleep problems and an urge to move, or complains of unpleasant sensations in the legs, the task force recommends that a series of questions should be asked. These questions are based on the RLS-Diagnostic Index (RLS-DI), which is a validated diagnostic algorithm combining essential and supportive diagnostic criteria of RLS [35]. The most important questions concern the urge to move the legs and the worsening of symptoms at rest. If a patient answers yes to three or more of these questions then the physician should question the patient about associated and supportive features (Table 1) of RLS that are the presence of RLS in the family, a positive response to dopaminergic therapy, and exclusion of other disorders (Table 3).

If the patient answers positively to one of the supportive/associated features, then it is likely that they have RLS.

**Figure 1:**
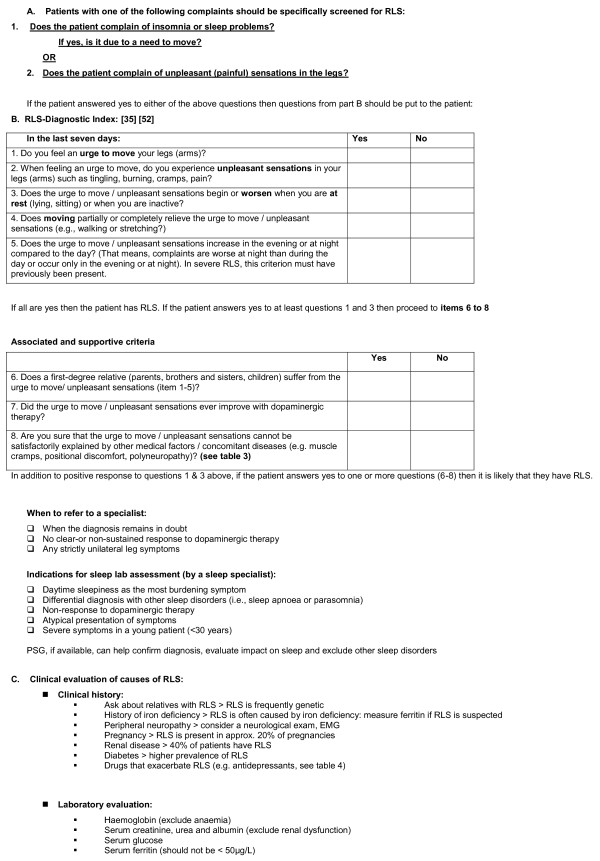
**Diagnostic algorithm**.

The diagnostic algorithm is detailed in Figure 1.

### General treatment considerations

#### A chronic disorder requiring long-term treatment

The natural clinical course of RLS varies between primary (idiopathic) and secondary (symptomatic) forms. Primary RLS tends to be chronic, with symptom severity increasing over time, this is especially the case in early-onset RLS [36], with many patients not developing daily RLS until the age of 40-60 years [37].

Patients with late-onset RLS often experience a more rapid progression of symptoms [38]. The remission of symptoms is possible in primary RLS, although it is difficult to know the course of RLS in mild or intermittent cases as patients often fail to consult their physician [39]. It is likely that patients with primary RLS will require treatment throughout their lives, and therefore need to be made aware of this before treatment initiation; possible side effects will also need to be discussed (Table 4). In comparison, secondary RLS might remit once the underlying condition (pregnancy, iron deficiency, chronic renal insufficiency) is resolved [40-42]. GPs, as well as patients should be made aware that the differentiation between primary and secondary RLS is somewhat arbitrary, as in many cases, iron deficiency is part of primary RLS and may never be completely resolved although repeatedly treated.

**Table 4:** Overview of treatments

Drug	Starting dose and maximum recommended dosage	Time to full effective therapeutic dose	Half-life	Side effects
Levodopa	50 mg 200 mg	At first dose	1.5-2 hours	Augmentation Rebound

Ropinirole	0.25 mg 4 mg	4-10 days	6 hours	Nausea, low blood pressure, dizziness, headache, nasal congestion

Pramipexole	0.125 mg 0.54 mg	At first dose	8-12 hours	Nausea, low blood pressure, dizziness, headache, nasal congestion

Rotigotine	1-3 mg patches	1 week	5-7 hours	Skin irritation, nausea, low blood pressure, dizziness, headache, nasal congestion

Pregabalin	25-300 mg	3-6 days	10 hrs	Sleepiness, dizziness, headache, fluid retention

Clonazepam	0.50 mg 2.0 mg	First dose: effect mainly on sleep	30-40 hours	Sleepiness, dizziness, morning drug hangover

Gabapentin	300 mg 2700 mg	3-6 days	5-7 hours	Sleepiness, dizziness, fluid retention

It is important to remember that RLS treatment so far is symptomatic, not preventive. Treatment improves the quality of life of the patient and it is therefore important for the physician to work closely with the patient in tailoring treatment to their individual needs and paying close attention to any symptom fluctuations. In addition, RLS treatment does not have a constant effect over the 24-hour period, as many RLS treatment options have a short half-life and should only be administered a few hours before symptoms begin in the evening. The main exception to this need is rotigotine, a 24-hr acting drug that is usually administered as a patch in the morning and does not need to be adjusted to the individual time of onset of symptoms (Table 4).

#### Exacerbators of RLS

Most patients who are diagnosed with RLS will have already tried and tested many non-pharmacological options by the time they seek medical attention such as activities that keep them concentrated, the avoidance of caffeine and alcohol, hot baths etc, so the task force decided not to discuss non-pharmacological treatment in this paper. However, there are a certain number of medications that are known to exacerbate RLS symptoms and their use should be reconsidered, these include antihistamines, dopamine antagonists, anti-nausea medications, antidepressants, serotonergic reuptake inhibitors, neuroleptics, beta-blockers, some anticonvulsants, and lithium (Table 5) [43].

**Table 5:** Drugs that may exacerbate RLS

Diphenhydramine (and other over the counter cold remedies)
Metoclopramide
Prochlorperazine
Chlordiazepoxide
Traditional antipsychotics (phenothiazines)
Atypical neuroleptics (olanzapine and risperidone)
Antidepressants (especially norepinehrine or selective serotonin reuptake inhibitors)
Anticonvulsants (zonisamide, phenytoin, methsuximide)
Antihistamines
Opiods

#### Drug dosages should be kept to a minimum

The drug dosages given to RLS patients should be kept to the strict minimum, and the maximum regulatory dose should never be exceeded (Table 4). It is important that physicians know that for the dopaminergic agents the doses required for RLS are far lower than those used to treat Parkinson's disease patients. The first-line treatments for RLS have not been approved in divided doses (i.e. dividing the full dose into two administrations, to cover evening and sleep, as opposed to dividing the dose during the day); whenever possible dividing doses should therefore be avoided in as far as that means increasing the total daily dosage. In some patients, however, a single dosage may not be sufficient for long-term treatment and these patients especially have to be carefully followed to keep the 24 h dosage low.

Treatment should be administered for a sufficient duration for an effect on symptoms to be seen before switching to a different drug. This however, depends on the individual drug (see Table 4). Caution should be exercised when increasing the treatment dosage, and continuous increases should be avoided as this can lead to a serious treatment-complication called augmentation (see below).

### When to treat?

#### Clinical significance

**Figure 2:**
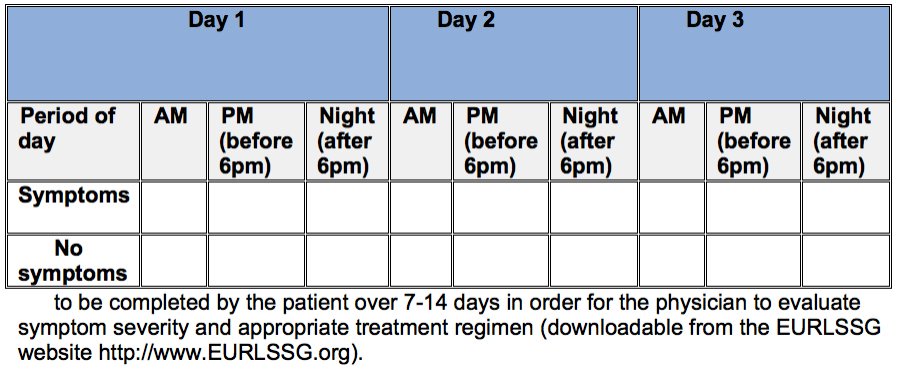
Symptom diary.

RLS should only be treated when it is clinically significant, that is, when symptoms impair the patient's quality of life, daytime functioning, social functioning or sleep. To facilitate the evaluation of RLS severity and to monitor treatment efficacy the task force recommends the use of a simple sleep diary that should be used for 7-14 days (see Figure 2 also available for download from the EURLSSG website http://www.eurlssg.org).

### How to treat

#### Categories of treatment and which drugs to use (for recommended doses see the treatment algorithm Figure 3)

**Figure 3:**
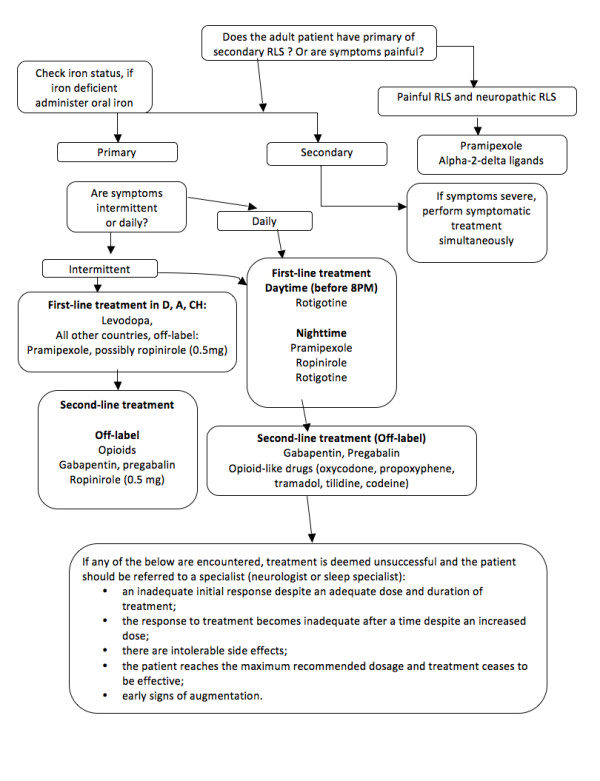
Treatment algorithm.

##### a) Intermittent vs. continuous

Patients with RLS are divided into different treatment categories: intermittent, daily and refractory.

Clinically significant intermittent RLS is present when symptoms do not occur frequently enough to require daily treatment. Although no treatments have been approved for intermittent RLS, the intermittent use of levodopa or pramipexole can be considered to be most appropriate if an off-label treatment is warranted. Other off-label treatment options include low-potency opioids, or if symptoms mainly disturb sleep, a hypnotic such as clonazepam, although its use is off-label. (see Figure 3 for more details).

Daily treatment is necessary for patients with moderate to severe RLS that has a negative impact on their lives either every day or on most days of the week. In such cases the dopamine agonists (pramipexole, ropinirole, and rotigotine) are the first-line treatment choice [8]. If symptoms occur at night, treatment can be initiated with a low dose of either pramipexole, ropinirole or rotigotine. However, in addition to nighttime symptoms, the patient might describe symptoms during the daytime. Such daytime symptoms are not uncommon and can particularly break through during immobilization or any other changes in lifestyle. Such cases should be treated preferentially with transdermal rotigotine due to its longer duration of action [44]. Second-line treatment consists of opioid-like drugs (e.g. tramadol, tilidine and codeine) but their use over the long-term could be problematic due to addiction issues [8]. Alpha-2-delta ligands (pregabalin, gabapentin and gabapentin enacarbil) are currently being examined in clinical trials and might constitute a promising alternative if their efficacy is confirmed in long-term trials [45,46].

Refractory RLS is daily RLS that has been unsuccessfully treated with two classes of drugs (one dopaminergic and one non-dopaminergic) at the correct dose and for an adequate length of time. Refractory RLS should be referred to the appropriate specialist and no longer be treated in the primary care clinic.

##### b) Primary vs. secondary

For primary RLS the physician should administer treatment either intermittently or continuously as detailed above.

Secondary RLS is often associated with iron deficiency, low serum ferritin values, pregnancy, end-stage renal disease (ESRD), rheumatoid arthritis, diabetes or with neurological disorders such as polyneuropathy, and various forms of spinal disorders. While laboratory tests are likely to be normal in primary RLS, in order to rule out or treat secondary RLS it is important to treat iron deficiency, which is implicated in both the onset of secondary RLS as well as in the severity of RLS [47,48], and is common during pregnancy and ESRD.

The task force recommends that hemoglobin, transferrin saturation and serum ferritin are evaluated in all RLS patients and that oral iron be administered to replenish iron when serum ferritin levels are < 50 μg/L. In those cases, iron substitution should be administered in parallel to other treatments [49]. In some cases, intravenous iron therapy can be an effective treatment.

Painful forms of RLS or any RLS associated to polyneuropathy (diabetes etc.) might respond well to alpha-2 delta agonists (pregabalin, gabapentin). Pramipexole has also been shown to improve painful symptoms in RLS patients [50].

For RLS in pregnancy and breast-feeding, only iron and folic acid can be recommended. There are no specific recommendations for the elderly (> 75 yrs). Children with RLS should be referred to an RLS expert.

##### c) Daytime symptoms?

RLS symptoms can occur during the day, and at least in one study, this has shown to occur in over 40% of the cases [6]. In such cases the task force recommends treatment with rotigotine, which is administered as a patch and provides therapeutic plasma levels over the entire 24-hr period. Extended release dopamine agonists are available for other indications but have not been approved for RLS. So far, no studies have been published on their use and potential advantages over standard immediate release forms in RLS.

#### 2. How long to treat

Unfortunately, at the present time, data are lacking concerning treatment duration. However, the task force recommends that treatment should be stopped in the following cases:

• On the patient's request;

• Following causal interventions (e.g. renal transplants);

• Periodically, e.g., every year for a few days if possible, to evaluate whether there are any spontaneous fluctuations in disease severity. This is not applicable for all patients especially those who are severely affected

#### 3. Treatment follow-up

Despite the existence of RLS severity rating scales, which are used in clinical trials, the task force agreed that such scales should not be used to initiate treatment. Although severity has been shown to correlate sufficiently with the patient's quality of life, this may depend upon the scale rather than the true clinical picture.

Principally, the task force recommends that the GP should see the RLS patient every six to twelve months for follow-up. In order to appropriately monitor treatment efficacy and non-responders, the use of a simple symptom diary is recommended (Figure 2). This diary will give an indication of the severity of the patient's symptoms and the effect of treatment, and will also enable the identification of augmentation.

### Treatment complications

#### When to refer to a specialist?

Patients should be referred to a specialist (either a sleep specialist or a neurologist) if treatment proves to be unsuccessful. The task force defines unsuccessful treatment as:

• an insufficient initial response despite an adequate dose and duration of treatment;

• the response to treatment becomes insufficient after a time despite an increased dose;

• there are intolerable side effects;

• the patient reaches the maximum recommended dosage and treatment ceases to be effective;

• augmentation develops;

• In general, children should not be treated at the primary care level.

#### Augmentation

Augmentation is the main complication of long-term dopaminergic treatment of RLS. It is characterized by an overall increase in RLS symptom severity which means that the symptoms appear earlier in the day, they occur quicker when the patient is at rest, and may spread to other body parts including the trunk and arms. The most effective way of preventing augmentation is to keep the dose of the dopaminergic medication as low as possible, ensuring that it does not exceed the dose recommended by regulatory authorities, and to prefer drugs with a long half-life/duration of action (see above). If augmentation is suspected then treatment should be changed from a dopaminergic to either a longer-acting dopaminergic or to a non-dopaminergic drug [51], except in the case of augmentation under levodopa when a switch to a dopamine agonist is recommended in the first instance. If this proves to be unsuccessful, referral to a specialist is recommended.

### When should augmentation be suspected?

Augmentation should be considered a possibility when

• any maintained increase in symptom severity despite appropriate treatment;

• any maintained increase in symptom severity following a dose increase, particularly if a dose reduction leads to an improvement in symptoms;

• any earlier onset of symptoms in the afternoon/evening;

• any spreading of symptoms to previously unaffected body parts;

• any shorter latency to symptom onset during the day when at rest.

## Discussion and Conclusion

RLS is a common condition that can present frequently in primary care setting. Due to the important consequences on quality of life, and the availability of treatment, it is important to identify such cases in primary care. RLS is probably one of the most easily manageable medical causes of insomnia, and thus, it is important that GPs become familiar with this disorder, it's diagnosis and management. Given the impact of RLS on quality of life and the marked therapeutic efficacy of current treatments in improving these symptoms, its early identification and treatment is highly relevant.

## Competing interests

Heike Benes has received honoraria for advisory boards or lectures from Boehringer Ingelheim, GlaxoSmithKline, Lundbeck, MSD and UCB Pharma.

Heiner Buschmann has reported no conflict of interest.

K. Ray Chaudhuri has received honoraria for symposium lectures and advisory boards from UCB, Abbott, Boehringer Ingelheim, Britannia, and GlaxoSmithKline; Academic grants from UCB, Brittannia, Abbott.

Diego Garcia-Borreguero has received honoraria for advisory boards or lectures from Boehringer Ingelheim, GlaxoSmithKline, UCB Pharma, Pfizer, Xenoport, Sanofi-Aventis, Jazz Pharma and MSD.

Birgit Högl has received honoraria for advisory boards, or lecutres from Boehringer Ingelheim, GlaxoSmithKline, UCB Pharma, Pfizer, Jazz, Sanofi-Aventis and Cephalon.

Ralf Kohnen has received honoraria from UCB, Pfizer and Axxonis for advisory board membership; UCB and Mundipharma for consultancy; and UCB and Axxonis for expert testimony.

Víctor Manuel González Rodríguez has received honoraria for advisory boards or lectures from Lilly, Pfizer, EISAI, Wyeth, GlaxoSmithKline, Lundbeck, Almirall and Janssen Cilag.

Giorgio Carlo Monti has reported no conflict of interest.

Karin Stiasny-Kolster has received honoraria for advisory boards or lectures from Boehringer Ingelheim, Orion, Mundipharma, Pfizer, Schwarz Pharma, Synosia and UCB Pharma.

Paul Stillman has reported no conflict of interest.

Claudia Trenkwalder has received honoraria for advisory boards or lectures from Boehringer Ingelheim GmbH, Cephalon, GlaxoSmithKline, Mundipharma, Orion Pharma, UCB/Schwarz Pharma, and Novartis.

Anne-Marie Williams has reported no conflict of interest.

Marco Zucconi has received honoraria for lectures from Boehringer Ingelheim GmbH and GlaxoSmithKline.

## Authors' contributions

DGB participated in the conception of the study, in its design, the acquisition, analysis and interpretation of data, and the writing and review of the manuscript. AMW participated in the in the conception of the study, in its design, the acquisition, analysis and interpretation of data, and the writing, review and coordination of the manuscript HK and RK participated in the design, the acquisition, analysis and interpretation of data, and the review of the manuscript. PS, HB, BH, KSK, CT and MZ were involved in the analysis of data, the drafting and review of the manuscript. KRC and VMG participated in the drafting and review of the manuscript. All authors read and approved the final manuscript.

## Pre-publication history

The pre-publication history for this paper can be accessed here:

http://www.biomedcentral.com/1471-2377/11/28/prepub
